# A High Throughput Cell-Based Screen Assay for LINE-1 ORF1p Expression Inhibitors Using the In-Cell Western Technique

**DOI:** 10.3389/fphar.2022.881938

**Published:** 2022-05-24

**Authors:** Yanni Kou, Shujie Wang, Yanjie Ma, Ning Zhang, Zixiong Zhang, Qian Liu, Yang Mao, Rui Zhou, Dongrong Yi, Ling Ma, Yongxin Zhang, Quanjie Li, Jing Wang, Jinhui Wang, Xile Zhou, Chunnian He, Jiwei Ding, Shan Cen, Xiaoyu Li

**Affiliations:** ^1^ Institute of Medicinal Biotechnology, Chinese Academy of Medical Sciences and Peking Union Medical College, Beijing, China; ^2^ Peking Union Medical College Hospital, Chinese Academy of Medical Sciences and Peking Union Medical College, Beijing, China; ^3^ Department of Colorectal Surgery, The First Affiliated Hospital, Zhejiang University, Hangzhou, China; ^4^ Institute of Medicinal Plant Development, Chinese Academy of Medical Science, Beijing, China

**Keywords:** LINE-1, in-cell western, anti-tumor, ORF1P, natural active compound

## Abstract

Long interspersed nuclear element 1 (LINE-1) is a dominant autonomous retrotransposon in human genomes which plays a role in affecting the structure and function of somatic genomes, resulting in human disorders including genetic disease and cancer. LINE-1 encoded ORF1p protein which possesses RNA-binding and nucleic acid chaperone activity, and interacts with LINE-1 RNA to form a ribonucleoprotein particle (RNP). ORF1p can be detected in many kinds of tumors and its overexpression has been regarded as a hallmark of histologically aggressive cancers. In this study, we developed an In-Cell Western (ICW) assay in T47D cells to screen the compounds which can decrease the expression of ORF1p. Using this assay, we screened 1,947 compounds from the natural products library of Target Mol and Selleckchem, among which three compounds, Hydroxyprogesterone, 2,2':5′,2″-Terthiophene and Ethynyl estradiol displayed potency in diminishing LINE-1 ORF1p expression level. Further mechanistic studies indicated the compounds act by affecting LINE-1 RNA transcription. Notably, we demonstrated that the compounds have an inhibitory effect on the proliferation of several lung and breast cancer cell lines. Taken together, we established a high throughput screening system for ORF1p expression inhibitors and the identified compounds provide some clues to the development of a novel anti-tumor therapeutic strategy by targeting ORF1p.

## Introduction

LINE-1 (Long interspersed nuclear element 1) is the only currently active autonomous retroelement in the human genome. LINE-1 sequences make up approximately ∼17% of the chromosome, among which ∼80–100 copies of human LINE-1 elements are estimated to be potentially active in the human individual (Luan et al., 1993; Lander et al., 2001; Cost et al., 2002). LINE-1 is about 6,000 nucleotides in length, belonging to non-LTR retrotransposon. It carries a 5′ untranslated region (UTR), 3′UTR with poly(A) tail, and three open reading frames named ORF1, ORF2, and ORF0. (Scott et al., 1987; Dombroski et al., 1991). ORF1p is a 40 kDa protein with RNA-binding and nucleic acid chaperone activity while ORF2p is 150 kDa protein with endonuclease and reverse transcriptase activities (Feng et al., 1996; Moran et al., 1996; Kulpa et al., 2006). Three ORF1p proteins intertwined throughout the length of their N-terminal coiled-coil to form a homotrimer. The central RNA recognition motifs (RRM) and C-terminal domains together form deep intervening clefts which are likely to interact with the backbone of single-stranded RNA. ORF1p and ORF2p bind to LINE-1 RNA in a cis-acting manner to form a ribonucleoprotein particle (RNP) (Kulpa et al., 2006). Then The LINE-1 RNP is imported into the nucleus, where LINE-1 RNA is reverse-transcribed and inserted into cellular DNA via a process known as “target-site priming reverse transcription (TPRT)” (Luan et al., 1993; Christensen et al., 2005). The third ORF, ORF0, which is expressed from an antisense promoter in the 5′ untranslated (UTR) of LINE-1, influences the retrotransposition process, but its exact function is still poorly understood (Denli et al., 2015). Furthermore, LINE-1 proteins also support the retrotransposition of some nonautonomous elements including Alu and SVA (Chen et al., 2005; Belancio et al., 2008).

The mobilization of active LINE-1 retrotransposons may cause chromosomal rearrangement, insertional mutations and genetic instability and subsequently induce deleterious genetic diseases and cancers (Kazazian et al., 1988; Groden et al., 1991; Miki et al., 1992; Fodde et al., 2001; Burns, 2017). Several lines of evidences indicated intragenic LINE-1 hypomethylation influence their host genes in cancer and many other aspects of genomic biology in an Argonaute 2 (AGO2)-dependent manner (Aporntewan et al., 2011; Wanichnopparat et al., 2013). Numerous disorders such as psychiatric conditions, autism, paranoid schizophrenia, Systemic lupus erythematosus (SLE) and other autoimmune diseases are associated with LINE-1 RNA pathogenesis. In addition, accumulated cytoplasmic LINE-1 cDNA induced by LINE-1 derepression has been reported to trigger IFN-I response which contributes to cellular senescence in aging and age-related diseases (De Cecco et al., 2019). ORF1p has been well characterized in human cancers perhaps because LINE-1 DNA produces ORF1p at a 1,000–10,000-fold higher level than ORF2p. Although LINE-1 ORF1p is barely detectable in human somatic cells, it can be easily detected in a variety of cancer types including breast cancers, ovarian cancers, prostate cancers, bladder cancers, lung cancers, colorectal cancers, and esophageal cancers (Rodić et al., 2014). Furthermore, it has been reported that, in some high-grade carcinomas, higher levels of nuclear L1 ORF1p protein were correlated with poorer clinical outcomes (Ting et al., 2011). Although the relationship between the high-level expression of LINE-1 ORF1p and the development of cancers remains to be determined, the LINE-1 ORF1p was gradually regarded as a hallmark of human cancers and exhibited a potential clinical application value (Ardeljan et al., 2017). More important, several cell-based studies revealed that LINE-1 dysfunction is mechanistically linked with therapy resistance in cancer cells. For instance, LINE-1 has been proposed to promote the overexpression of androgen receptor (AR) as well as create splice variants of the AR genes by inserting into the host genome, and subsequently lead to the activation of several pathways that increase cell proliferation (Briggs et al., 2018). In addition, a study reported that LINE-1 ORF1p physically interacts with AR and modulate AR cytoplasm/nucleus translocation as well as promotes the proliferation of human prostate carcinoma cells in both ligand-dependent and independent manners (Lu et al., 2013). Another example from Reyes-Reyes et al. has shown LINE-1 plays a direct role in epithelial to mesenchymal transition (EMT) and promotes drug resistance to sunitinib maleate even without the RT activity of ORF2p (Reyes-Reyes et al., 2017). Likewise, Feng et al. reported ORF1p promotes HepG2 proliferation and increases the drug resistance of several chemotherapeutic drugs including epirubicin, cisplatin, and paclitaxel (Feng et al., 2013). Collectively, these evidences indicated ORF1p might be a promising therapeutic target in cancers, especially for chemotherapeutic resistance. Thus, it is reasonable to find small molecular inhibitors of ORF1p and conduct a proof-of-concept study to provide new insights into the rationale of ORF1 inhibitors in cancer therapy. Therefore, developing high-throughput assays to find the compounds that inhibit ORF1p expression in cancer cells might be a useful tool to investigate this issue.

The In-Cell Western (ICW) Assay, which is also called cytoblots or cell-based ELISA, is a quantitative immunofluorescence assay performed in 96 or 384 well microplates. ICW combines the specificity of immunoblotting with the replicability and throughput of ELISA. In this study, we developed a robust and reliable ICW assay to screen compounds that can diminish the expression of LINE-1 ORF1p. Using our established assay, we identified three small molecular compounds which downregulate ORF1p expression with the EC_50_ of 37.03, 32,31, and 12.37 μM respectively. Further mechanistic studies revealed the compounds act through inhibiting LINE-1 RNA transcription. The compounds can inhibit the cell proliferation of several carcinoma cells in a dose-dependent manner. Altogether, our study provides a novel and robust assay for finding new molecules that inhibit LINE-1 ORF1p expression or LINE-1 transposition, which might be a novel tool easy to manipulate to study the impact of LINE-1 transposition in any cellular context. Notably, the cellular proliferation profiles of several cancer cell lines treated with ORF1p expression inhibitors indicated its potential application as a novel therapeutic target in cancer therapy in future.

## Results

### Establishment of an In-Cell Western Based Screening Assay for LINE-1 ORF1p Expression Inhibitor Screen

**FIGURE 1:**
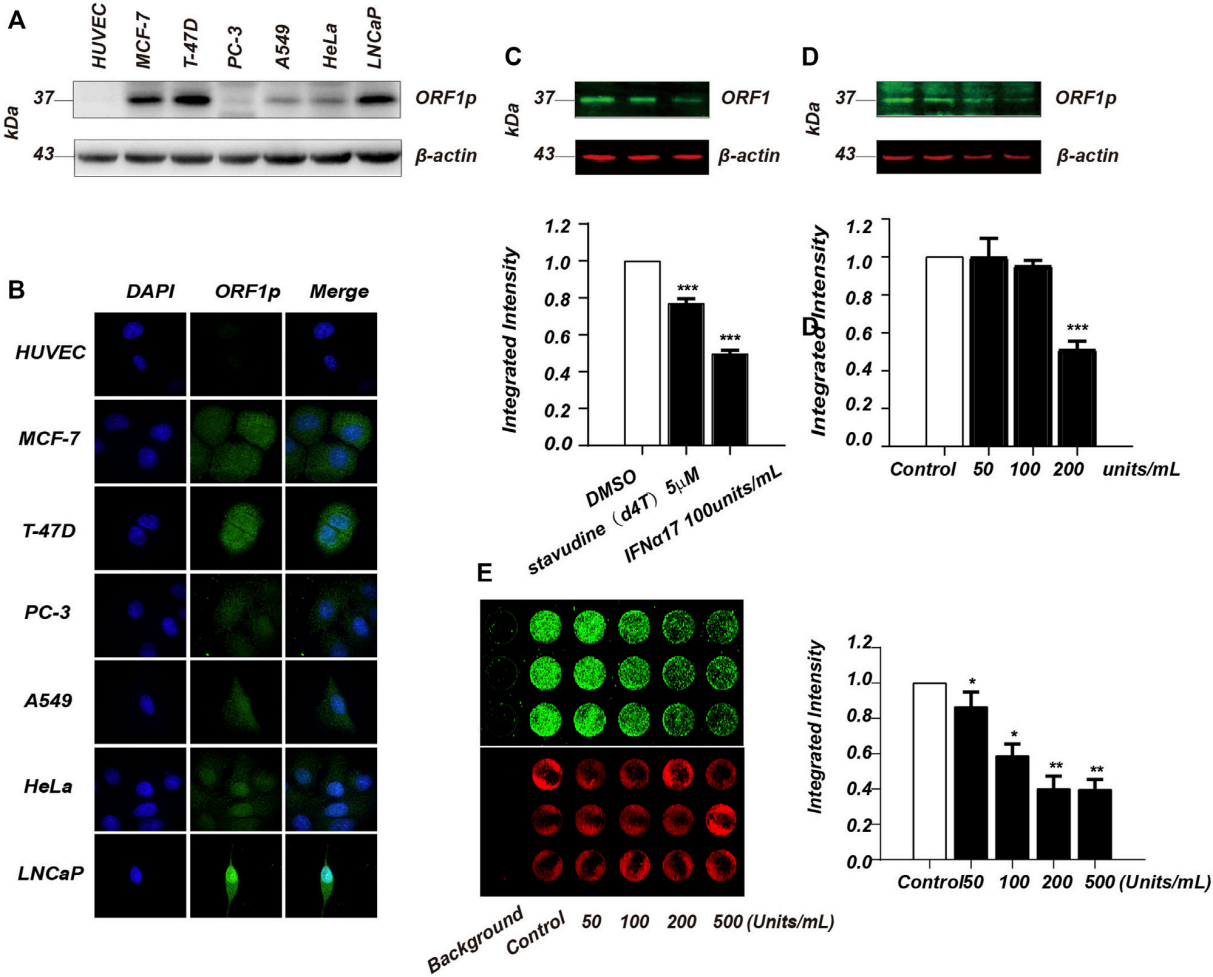
Establishment of ICW-based screening assay for LINE-1 ORF1p inhibitor screen. **(A)**, Immunoblotting analysis of LINE-1 ORF1p expression in six cancer cell lines including MCF7, T-47D, PC-3, A549, HeLa and LNCaP. **(B)**, Immunofluorescence assay of LINE-1 ORF1p expression in the cancer cell lines indicated. **(C)**, Immunoblotting analysis evaluation of the inhibitory activity of IFNα17 and d4T upon LINE-1 ORF1p in T-47D cells. **(D)**, The T-47D cells were treated with increasing doses of IFNα17 (50, 100, 200, and 500U) and LINE-1 ORF1p expression level was detected 48 h later by immunoblotting analysis. **(E)**, ICW assay analysis of LINE-1 ORF1p expression level in T-47D cells after being treated by increasing doses of IFNα17. All experiments were performed three times and statistical significance was determined using Students two-tailed *t*-test. *p < 0.05, **p < 0.01.

To develop an ICW based screen assay, we first set out to find a cell line that has a high expression level of LINE-1 ORF1p. Although LINE-1 ORF1p expression is hardly detectable in normal somatic cells, its overexpression has been previously found in many cancer tissues (Rodić et al., 2014). We therefore evaluated LINE-1 ORF1p expression level in several cancer cell lines by immunoblotting analysis. Human Umbilical Vein Endothelial Cells (HUVEC) which do not express ORF1p was used as a negative control. We evaluated LINE-1 ORF1p expression level in six cancer cell lines including MCF-7, T-47D, PC-3, A549, HeLa and LNCaP by immunoblotting assay (Figure 1A), and found the ORF1p expression level varies substantially in these cancer cell lines. Among them, MCF7, T-47D and LNCaP showed higher ORF1p expression levels than other cell lines. This result was also confirmed by immunofluorescence assay (Figure 1B). Considering that T-47D has excellent adhesion properties to polystyrene plates and was relatively easy to cultivate, we chose T-47D to develop the ICW assay.

It was previously reported that IFN-α and several nucleoside analogs such as d4T, AZT and 3 TC all can suppress LINE-1 retrotransposition through different mechanisms (Jones et al., 2008; De Cecco et al., 2019). To find a positive control in our ICW assay, we therefore chose IFNα17 and d4T to detect their inhibitory activity against LINE-1 ORF1p expression. The immunoblotting result showed that IFNα17 can obviously decrease ORF1p level in a dose-dependent manner, as opposed to d4T which has little effect on LINE-1 ORF1p expression (Figure 1C, D). As expected IFNα17 also decreased fluorescence value in a dose-dependent way (Figure 1E), suggesting that IFNα17 can serve as a positive control in inhibiting LINE-1 ORF1p expression level in the ICW assay.

### Optimization of the ICW Assay for LINE-1 ORF1p Expression Inhibitor Screening

**FIGURE 2:**
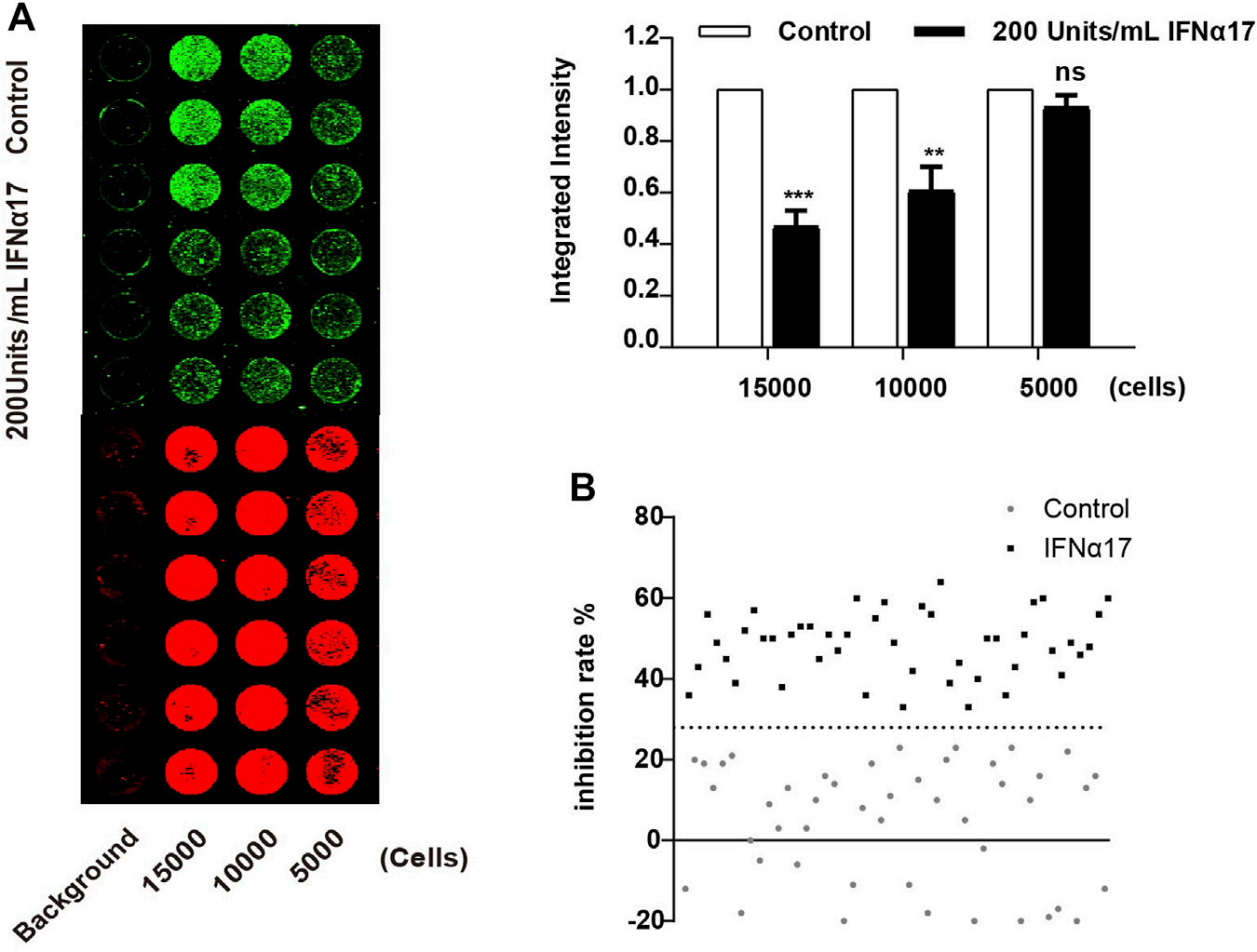
Optimization of the ICW assay for LINE-1 ORF1p expression inhibitor screening. **(A)**, Optimization of the cell density seeded in the 96 well plate. T-47D cells were seeded in 96 well plate at the concentration of 5,000, 10,000 and 15,000 cells per well, and then treated with IFNα17 (200U/ml), 48 h later, ICW assay were performed (left panel), the relative ORF1p expression level was calculated by the ratio of the integrated intensity value of IFN treated cell to that of DMSO treated cells (right panel). **(B)**, Determination of Z′ factor of the ICW based screening assay. One-half plate of T-47D cells was incubated with IFNα17 (200 units/mL) or PBS as a vehicle control for 48 h. Then the plate was scanned with the Odyssey system (LI-COR), the integrated fluorescence intensities and Z′ factor was calculated. All experiments were performed three times and statistical significance was determined using Students two-tailed *t*-test. *p < 0.05, **p < 0.01.

To improve the ICW assay sensitivity, we first optimized the cell density planked in the 96 well plate. We evaluated three different cell densities (15,000, 10,000 and 5,000 cells per well), and the result showed the detection sensitivity peaked at 15,000 cells per well (Figure 2A). Next, we assessed the key performance parameters of the ICW assay to evaluate its performance and robustness. The Z’ factor of the assay was 0.55, which meet the criteria required for High-Throughput Screen (HTS) assays (Iversen et al., 2006) (Figure 2B). Together, we have developed a robust and sensitive ICW-based assay which is further shown to be suitable for discovering LINE-1 ORF1p expression inhibitors.

### Screening Natural Products Library Using the ICW Assay

**FIGURE 3:**
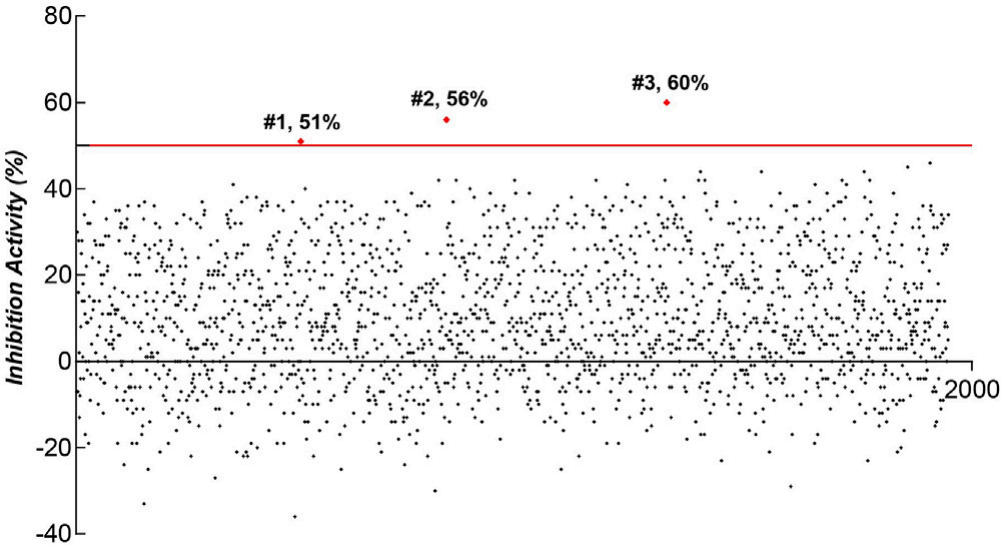
ICW-based assay screen of natural products for the compounds that inhibit LINE-1 ORF1p expression in T47D cells.

Natural compounds represent an extraordinary inventory of high structural diversity that can render them as privileged templates for designing novel medications at present time. Therefore, we screened the natural compounds library of Target Mol and Selleckchem to identify possible lead molecules against LINE-1 ORF1p using our cell-based ICW assay. 1,947 compounds were screened and three hits, #1 (Hydroxyprogesterone), #2 (2,2':5′,2″-Terthiophene) and #3 (Ethynyl estradiol) showed inhibitory activity over one-fold and were selected for further analysis (Figure 3).

### Evaluation of the Anti-ORF1p Activity of the Positive Natural Products

**FIGURE 4:**
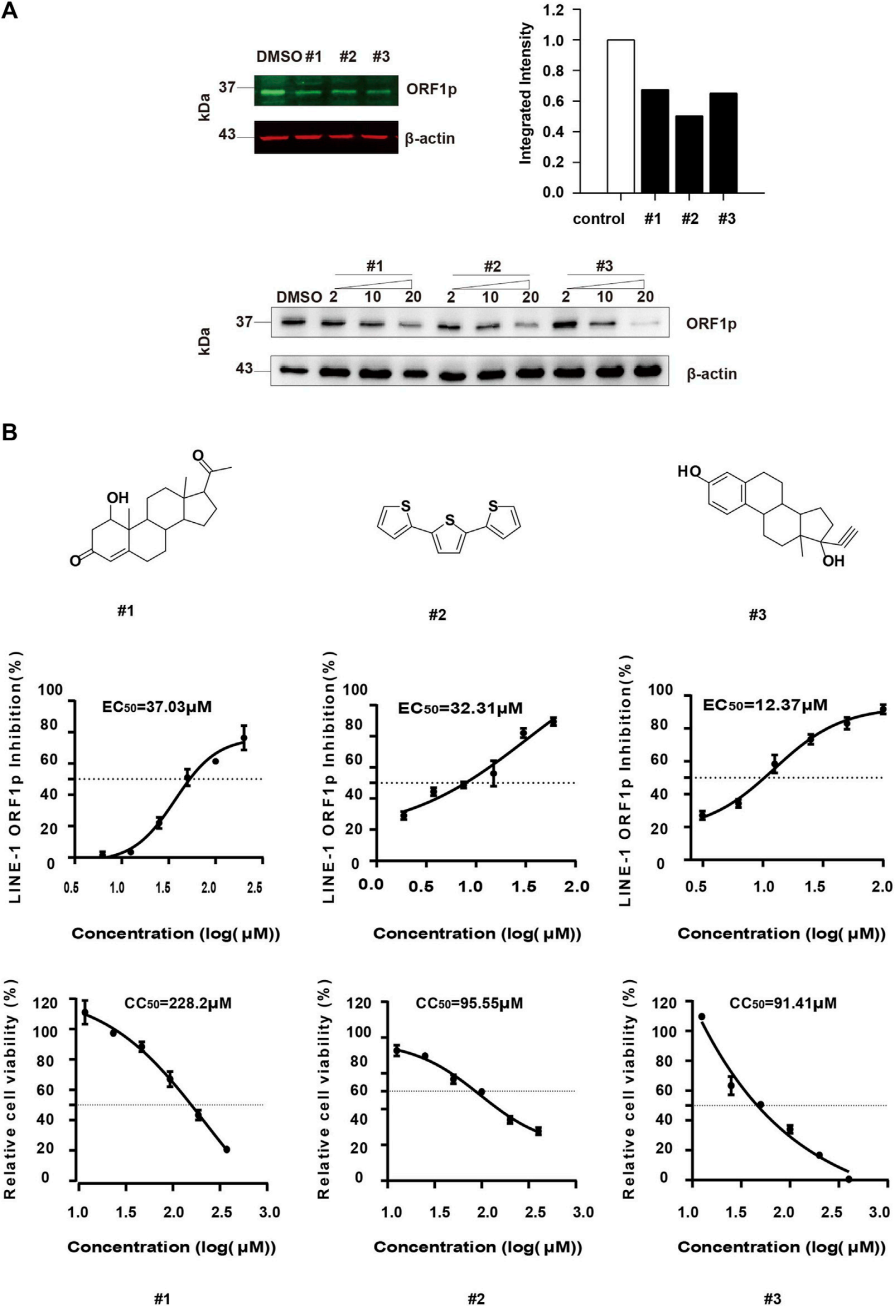
Evaluation of the anti-ORF1p activity of the selected hits. **(A)**, The T-47D cells were treated with #1, #2 and #3 at different concentrations, and 48 h later, the ORF1p expression level was detected by immunoblotting assay. **(B)**, Determination of the EC50 and CC50 of the compounds. T-47D cells (15000/wells) were seeded in 96-well plates, and then treated with the serially diluted compound. After 24 h incubation, the LINE-1 ORF1p expression level was detected by ICW. To assess cell viability, T-47D cells (1000/wells) were seeded in 96-well plates, and treated with these inhibitors as indicated above. The CC50 values were measured. Results shown are the average of three independent experiments.

We further verified the inhibitory activity of the three compounds against ORF1p by Odyssey Western blot (Figure 4A upper panel) and immunoblotting assay (Figure 4A lower panel). The result showed that all of the three compounds can diminish the LINE-1 ORF1p level in a dose dependent manner, thus confirming our screening results by ICW assay (Figure 4A). Next, we determined the EC_50_ value of each compound. The compound, #1, #2, and #3, displayed potency in diminishing LINE-1 ORF1p expression level with EC_50_ values of 37.03μM, 32.31μM and 12.37μM, respectively (Figure 4B). In addition, the CC_50_ values of the three compounds were 228.2μM, 95.55μM, and 91.41 μM respectively (Figure 4C), with the therapeutic index (TI) value of 6.16, 2.95, and 7.38. Collectively, the compounds showed considerable inhibitory activity against LINE-1 ORF1p expression and relatively low cytotoxicity.

### The Effect of the Compounds on LINE-1 RNA Synthesis

**FIGURE 5:**
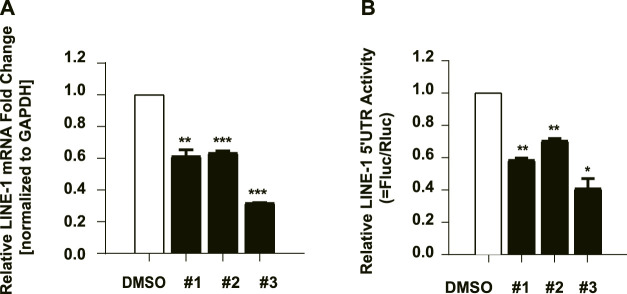
The effect of the compounds on LINE-1 RNA synthesis. **(A)**, The effect of the compounds on the LINE-1 RNA level in T-47D cells. T-47D cells were treated with #1, #2 and #3 (10 μM) respectively, and 48 h later, the LINE-1 mRNA level was quantified using RT-qPCR. **(B)**, 293 T cells were transfected with L1-FL plasmid, and then treated with the indicated compound. The firefly luciferase activity driven by the LINE-1 5′-UTR promoter was measured. pGL3-basic which contains the firefly luciferase gene that lacks a promoter at its 5′ end serves as a control vector. Firefly luciferase activity in the pGL3-basic transfected cells was measured to reflect the basal expression of the firefly luciferase gene. Data represent the mean ± SD of three independent experiments. *p*-values were calculated using a standard Student’s t-test. *p < 0.05, **p < 0.01, ***p < 0.001.

To decipher the underlying mechanism of the anti-ORF1p activity of these compounds, we further investigate whether the inhibitory activity of these compounds is derived from their effect on the LINE-1 RNA level. We therefore next tested whether these compounds affected LINE-1 RNA level using RT-qPCR. T-47D cells were treated with the compounds at 10μM, and the LINE-1 RNA level was detected 48 h later. All of the three compounds diminished the LINE-1 RNA level compared to the DMSO negative control (Figure 5A), suggesting the inhibitory activity of the three compounds against ORF1p is ascribed to their ability to affect the LINE-1 RNA level. We also tested whether IFNα17 affected LINE-1 RNA level using RT-qPCR. T-47D cells were treated with IFNα17(200Units/mL), and the LINE-1 RNA level was detected 48 h later. The result showed that IFNα17 suppressed the LINE-1 RNA level by over 60% at the concentration of 200 Units/mL (Supplementary Figure S1A).

Given that the decreased LINE-1 mRNA level might be derived from a lower transcriptional level or poorer stability. We next cloned the 5’ UTR region of LINE-1 which contains the internal promoter for its RNA transcription (Swergold, 1990) into a pGL3 promoter upstream of a luciferase reporter gene (L1-FL) (Li et al., 2013)**,** and used it to detect the effects of these compounds upon the LINE-1 promoter in 293 T cells. The result showed that #3 suppressed the luciferase value by over 60% at the concentration of 10 μM, while #1 and #2 only exhibited mild effects on the luciferase value (Figure 5B) which was similar to IFNα17 (Supplementary Figure S1B). these data collectively indicated compound #3 inhibited LINE-1 RNA level by suppressing LINE-1 RNA transcriptional initiation, while #1 and #2 diminished LINE-1 RNA level might through other mechanisms that need further investigation. Furthermore, in order to exclude out the decreased L1-Fluc activity was derived from the effects on the luciferase expression itself, we used the pcDNA4-Luc plasmid which encodes a luciferase reporter as a control by transfecting it into HeLa cells followed with the treatment of the compounds and IFNα17. The data showed that these compounds had no effect on the luciferase gene itself (Supplementary Figure S2).

### The Effect of the Compounds on LINE-1 Retrotransposition

**FIGURE 6:**
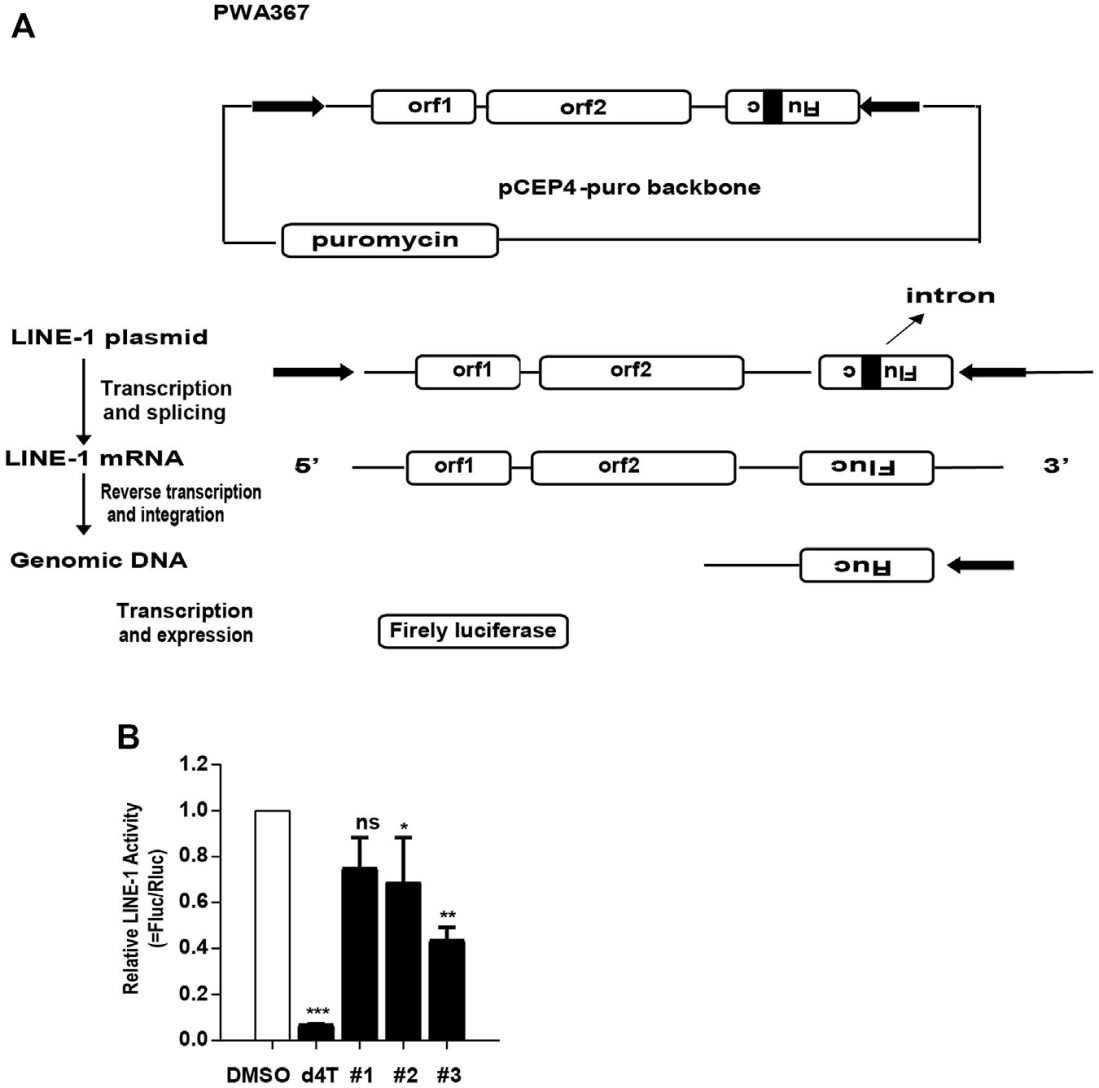
The effect of the compounds on LINE-1 retrotransposition. **(A)**, Illustration of the pWA367 reporter. Following transcription from the P1 promoter of LINE-1, the intron in the Fluc reporter gene is removed. The intronless mRNA is then reverse transcribed into cDNA that is able to produce a functional Fluc gene reporter mRNA. **(B)**, The effect of the compounds on LINE-1 mobilization. HeLa cells were transfected with pWA367 reporter DNA and then treated with #1, #2 and #3 (10 μM) respectively. The Fluc/Rluc represent LINE-1 mobilization activity. The data from three independent experiments were summarized in the bar graph. *p*-values were calculated using a standard Student’s t-test. *p < 0.05, **p < 0.01, ***p < 0.001, ns, not significant.

Given that the compounds diminish the LINE-1 ORF1p expression level and LINE-1 mRNA level, we want to figure out whether these compounds also inhibit LINE-1 retrotransposition. Using d4T as a positive control, we evaluated the effect of these compounds upon the LINE-1 transposition using a dual-luciferase scheme. This assay used a pWA367 reporter construct that has an intron inserted in the Fluc reporter gene in the opposite direction from the LINE-1 coding sequence (Xie et al., 2011), (Figure 6A). An intron sequence has been inserted in the same direction with the LINE-1 coding sequence, which ensures that the Fluc reporter gene will only be produced from the reverse transcribed LINE-1 DNA with the intron removed during RNA splicing. We transfected the pWA367 reporter plasmid into HeLa cells and treated the cells with the compounds during the retrotransposition assay. The results indicate that all of the three compounds can inhibit LINE-1 transposition, and 3# showed the best potent activity among them (Figure 6B).

### ORF1p Expression Inhibitors Repress the Proliferation of Several Carcinoma Cells

**FIGURE 7:**
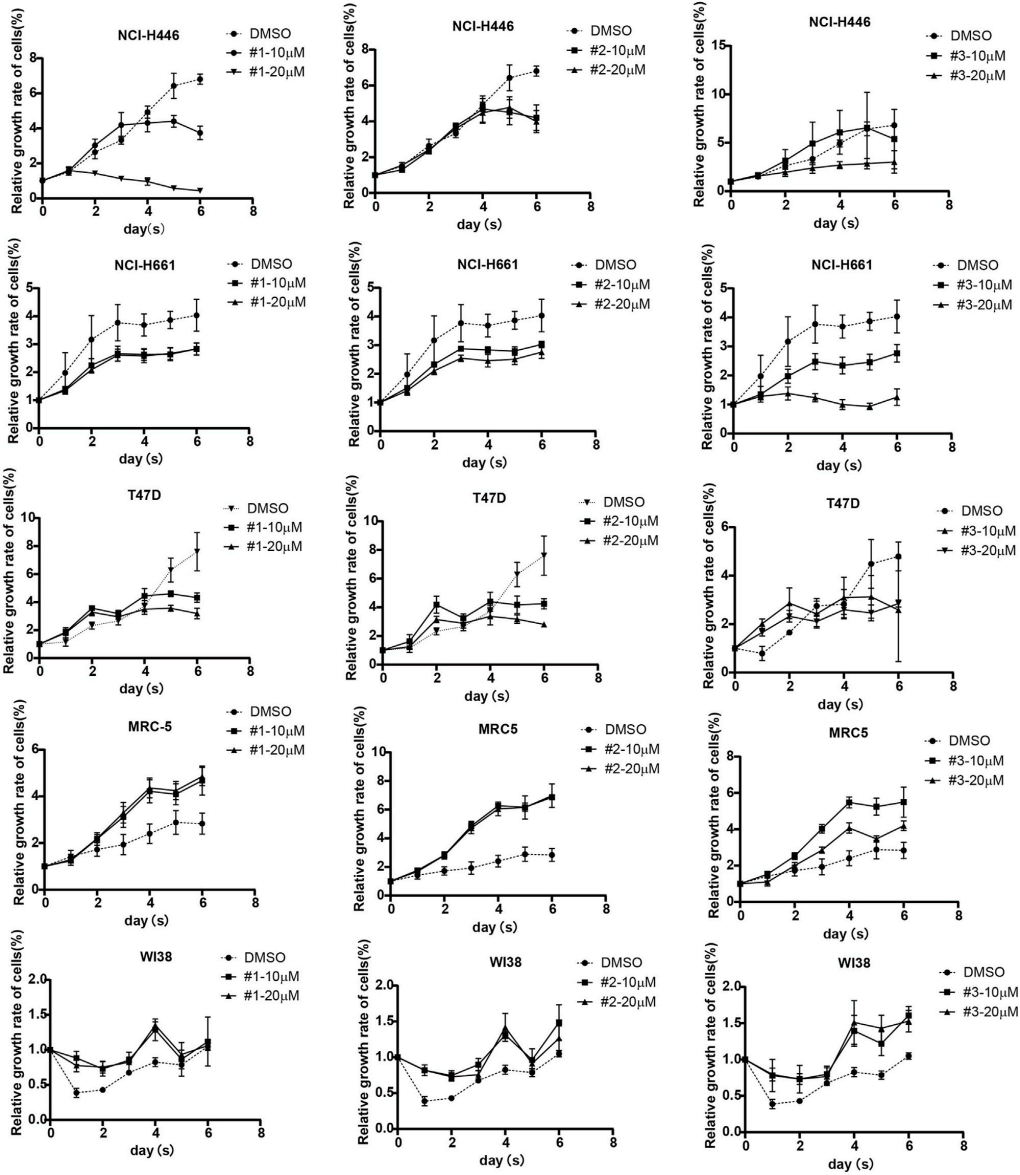
The effects of the compounds on the proliferation of carcinoma cells. Three carcinoma cells including NCl-446, NCl-661 and T47D as well as MRC-5 and WI38 cell lines as two negative controls were treated with compound #1, #2 and #3 at the concentration of 10 μM or 20 μM or DMSO as a, cell viability is measured for a consecutive 6 days by a CCK8 assay.

Several lines of evidence have indicated ORF1p is closely associated with cancer cell proliferation and increases drug resistance of antineoplastic agents (Feng et al., 2013; Ardeljan et al., 2017; Chen et al., 2018; Whongsiri et al., 2018; Budania et al., 2020), we went on to test whether the three compounds we identified have an impact on the proliferation of carcinoma cells. we took advantage of a lung cancer cell line (NCl-H661), a non-small-cell lung adenocarcinoma (NSCLC) cell line (NCl-H446), and one breast carcinoma cell line (T47D) reserved on our lab, as well as a human lung fibroblast cell line MRC5 and WI38 as a negative control. The effect of each compound was tested at two concentrations, 10 μM and 20 μM using a CCK8 assay measured during 6 consecutive days. It is interesting to find that the growth rate of NCl-H661 was markedly decreased upon the treatment of all three compounds, compared with controls treated with DMSO (Figure 7). Interestingly, the proliferation kinetics was different in T47D and NCl-H446 cells compared with that of NCl-661 cells, the inhibitory effects of the compounds seem to begin to work just from the 5^th^ day. In contrast, the growth rate of MRC-5 and WI38 cells was not inhibited by the three compounds confirming the concentrations we used were not sufficient to induce cellular cytotoxicity. At the same time, we verified the inhibition of ORF1 protein expression by these three compounds in NCI-H446, NCI-H661, T47D and MRC-5 cells. The decreased ORF1 expression was correlated with the inhibition of cell proliferation (Supplementary Figure S3). In addition, we also test whether IFNα17 was able to repress carcinoma cell proliferation in an ORF1p-dependent manner. Three carcinoma cell lines NCl-H446, NCl-H661 and T47D as well as MRC-5 cells as a negative control were treated with IFN α17 and the cellular proliferation was measured by a CCK8 assay. The results showed IFNa17 significantly decrease the proliferation of NCl-446 and NCl-661. With respect to T47D, the inhibition became obvious at a high concentration of IFNa17 (500Units/mL). (Supplementary Figure S4). Collectively, these data demonstrated that the compounds have potential anti-tumor effects by inhibiting the propagation of carcinoma cells.

## Discussion

There is increasing interest in deciphering the role of LINE-1 and its encoding proteins in the development of kinds of human disorders, and inhibiting its structural protein, ORF1p is a straightforward and efficient tool to investigate this issue. Thus, in the present study we develop an efficient high-throughput ICW assay for LINE-1 ORF1p expression inhibitor screening, which provides a proof-of-concept of implementing the same strategy to develop other original new drugs. The screen system was proved to be simple, rapid, robust and inexpensive.

Indeed, several studies have already focused on the nucleotide analog against RT activity of ORF2p and demonstrated targeting RT of ORF2p could inhibit LINE-1 retrotransposition and inhibited cancer cell line propagation (Carlini et al., 2010; Banuelos-Sanchez et al., 2019; Bellisai et al., 2020). To cite a few, Guillermo BS et al. reported GBS-149 is a potent inhibitor of LINE-1 retrotransposition by testing 33 nucleotide analogs (Banuelos-Sanchez et al., 2019). In addition, Cristina B et al. showed efavirenz (EFV) and SPV122.2, two nonnucleoside inhibitors against LINE-1, reduce proliferation and promote differentiation of cancer cells, concomitant with a global reprogramming of transcriptional profiles (Bellisai et al., 2020). Similarly, another study reported Abacavir (ABC), a nucleoside reverse transcription inhibitor (NRTI) elicited up-regulation of LINE-1 transcription, induced antiproliferative activity and triggered senescence in prostate cancer cells (Carlini et al., 2010). However, limited studies are focusing on potential ORF1p expression inhibitors up to now. The rationale of our study is based on two facts: 1) LINE-1 plays a direct role in the tumorigenesis and promotes drug resistance to anticancer drugs even without the RT activity of ORF2p (Reyes-Reyes et al., 2017) indicating a possible independent role of ORF1p in driving tumors, 2) ORF1p is transcribed from a bicistronic LINE-1 mRNA which far exceeds ORF2p in number and regarded as a hallmark of cancers. Thus, it is imperative to find potent non-toxic ORF1p expression inhibitors.

Taking advantage of our established assay, we identify three potent compounds targeting ORF1p expression, which are the first small-molecular inhibitors of ORF1p expression, to the best of our knowledge. They might be demonstrated a robust tool in the future to study the association of LINE-1 activity with a series of LINE-1-related disorders, such as Aicardi-Goutieres syndrome (AGS) and cancers, using a loss-of-function approach. Importantly, mechanistic studies indicated all three compounds diminish LINE-1 RNA level. Compound 3# exhibited a direct suppression on 5′UTR of LINE-1. Based on these observations, we propose they might interfere with the transcriptional initiation by binding to key factors necessary for LINE-1 transcription or impede the transcription elongation, which merits further investigation. Notably, both compounds 1# and 3# belong to steroids whereas compound 2# has a distinct chemical structure. The skeleton of compounds 1# and 3# resembles that of progesterone and estradiol respectively. The compound 1# carries a hydroxy substituent at position 1 and the compound 3# has an acetenyl substituent at position 17, which might confer altered bioactivities from the progesterone and estradiol. A research group investigated the association of hormonal levels in each phase of menstruation and genes containing LINE-1 expression (Chaiwongwatanakul et al., 2016). They revealed that high levels of estrogen (E2) in the proliferative phase (PR) might function to down-regulate genes containing LINE-1 expression. In contrast, high levels of progesterone may initiate up-regulated genes containing LINE-1. Our findings corroborate their previous conclusions indicating an association of sex hormones and LINE-1 expression regulation, and it would be interesting to dissect the molecular details about how sex steroids regulate intragenic LINE-1 expression in future. Sex hormones are involved in numerous biological systems including neuroendocrine, vascular, skeletal and immune systems (Hamilton et al., 2017). The mode by which sex hormones regulate gene transcription and expression has been extensively studied (Chan et al., 2000; Wang et al., 2000; Liu et al., 2001; Yie et al., 2006; Yin et al., 2007; Torrens-Mas et al., 2020). For instance, a study reported that 17beta-estradiol could regulate mRNA expression for specific dopamine and 5-HT receptors in a region-specific manner (Zhou et al., 2002). Similarly, SHANK expression has been reported to be influenced by sex hormones leading to a sex-differential expression, which might explain the sex bias in autism spectrum disorders (Berkel et al., 2018). Interestingly, female and male sex hormones have different impact on innate and adaptive immune response, which render women more predisposed to suffering from autoimmune diseases than men (Taneja, 2018). Considering that autoimmune diseases and neuro-disorders are closely associated with LINE-1 mobilization, it is of notably high-value to study if any “cross-talks” in signaling pathways exist between sex-hormones biased diseases (i.e., autism, autoimmune diseases) and LINE-1-induced pathologies.

Interestingly, our result showed that d4T did not affect LINE-1 ORF1p expression level in T-47D cells but inhibited LINE-1 retrotransposition. It is reasonable, for LINE-1 is undergone a reverse transcription process analogous to that of exogenous retroviruses, which is orchestrated by LINE-1 ORF2p, and, d4T is a reverse transcriptase inhibitor that belongs to nucleos(t)ide analogs (NAs). It imped the reverse transcription process by being incorporated into the nascent cDNA, thus terminating the cDNA synthesis. However, it does not affect the LINE-1 RNA transcription from the sequences that have been already integrated into the genome.

The cell proliferation assay implies the three compounds inhibited several cancer cell lines efficiently under the 0% toxicity concentration (TC_0_). However, not all the cancer cell lines tested showed a remarkable delayed progression under the treatment of the three compounds. It is probably due to that different cancer cell lines induce the malignancy through different mechanisms. NCl-H661 which is a lung cancer cell line showed high susceptibility to the three compounds, while another NSCLC cell line NCl-H446 and a breast cell line T47D were less susceptible to the compounds than NCl-H661 was. The different proliferation kinetics might be ascribed to the function and expression level of ORF1p in different types of carcinoma cells. Intriguingly, we also noticed the three compounds increased the cellular proliferation of MRC-5 which is a type of fibroblasts derived from normal lung tissue. Some literature has shown female sex hormones have certain effects on the cellular proliferation of fibroblasts. For instance, Ning L et al. found that estrogen stimulation enhanced the proliferation activity of fibroblasts (Luo et al., 2014). Estrogen receptors are present in fibroblast-like synoviocytes and estrogen, as well as progesterone, exert an effect on the fibroblast-like synoviocyte phenotypic changes (Khalkhali-Ellis et al., 2000). Notably, several steroidal molecules have been widely used for anti-tumor therapies. For instance, medroxyprogesterone is an anti-cancer drug in clinical practice with the indications of mammary carcinomas and endometrial carcinoma. The chemical structure of compound 1# and medroxyprogesterone is different only in the hydroxyl substituent position. Nevertheless, we could not exclude that the compounds have some unintended effects on other physiological processes, extensive pharmacology and toxicology studies should be carried out to determine its druggability for therapeutical purposes.

Usually, the screen system that inhibits LINE-1 retrotransposition might target the LINE-1 ORF2p protein that processes the reverse transcriptase activity. Our result indicates that inhibition of LINE-1 ORF1p expression can also affect the retrotransposition of the LINE-1 and inhibits the proliferation of cancer cell lines. These results collectively demonstrate that the ICW assay targeting LINE-1 ORF1p can also be used to screen novel compounds that inhibit LINE-1 transposition More importantly, we provide novel insights into the potential of ORF1p expression inhibitors in anti-tumor therapeutics.

## Materials and Methods

### Compounds

Two drug libraries-the natural compound library (Target Mol) and the natural product library (Selleck)-that together comprised 1,947 compounds were used.

### Cell Culture

HUVEC, T-47D, PC-3, A549, HeLa, LNCaP and HEK293 T cells were purchased from American Type Culture Collection (ATCC). MCF-7 cells were kindly provided by Stem Cell Bank, Chinese Academy of Sciences. HUVEC, A549, MRC-5, HEK293 T cells were cultured in Dulbecco’s modified Eagle’s medium (DMEM; Thermo Fisher Scientific) with 10% Fetal Bovine Serum (FBS; Gibco) at 37 C in a 5% CO_2_ incubator, T-47D, WI-38 and LNCaP cells were cultured in Roswell Park Memorial Institute−1640 (RPMI-1640; Thermo Fisher Scientific) with 10% Fetal Bovine Serum (FBS; Gibco) at 37 C in a 5% CO_2_ incubator. NCl-H446, NCl-H661, MCF-7 and HeLa cells were cultured in Minimum Essential Medium (MEM; Thermo Fisher Scientific) with 10% Fetal Bovine Serum (FBS; Gibco) at 37 C in a 5% CO_2_ incubator, and PC-3 cells were cultured in Ham’s F-12K (Kaighn’s) Medium (F-12K; Thermo Fisher Scientific) with 10% Fetal Bovine Serum (FBS; Gibco) at 37 C in a 5% CO_2_ incubator.

### Plasmid DNA and Antibodies

pWA367, containing an L1RP vector was provided by Dr. Wenfeng AN (South Dakota State University, USA) (Xie et al., 2011). The plasmid L1-FL was generated by inserting the 670 nt LINE-1 5′-UTR sequences into the pGL3-Basic vector (Promega) upstream of the luciferase reporter. pcDNA4-luc was generated by inserting open reading frame of the luciferase gene into pcDNA4 at the HindIII and EcoRI sites. HEK293 T and HeLa cells were transfected using Invitrogen™ Lipofectamine™ 2000 Transfection Reagent, according to the manufacturer’s instructions.

Anti-flag antibody (mouse) and anti-β-actin antibody (mouse) were purchased from Sigma-Aldrich, and ORF1p antibody (rabbit) is a gift from Dr. Guo Fei’s Lab.

### Western Blotting

Cells were collected and lysed with NP-40 buffer (Beyotime). The protein lysis was boiled for 20 min at 100 C in SDS-PAGE loading buffer. Equal amounts of cell lysate were 10% polyacrylamide-SDS gels. Proteins were transferred onto a PVDF membrane, blocked with 5% skim milk, and probed with anti-ORF1p antibody (diluted 1:1000) and anti-β-actin antibody (diluted 1:5,000) at 4°C overnight. After four wash steps with PBS plus 0.1% Tween 20 (PBST), the membrane was incubated with 1:5,000 dilution of HRP-conjugated goat-anti-mouse, HRP-conjugated goat-anti-rabbit, IRDye^®^ 800CW Goat anti-Rabbit 926–32,211 (1:10,000, LI-COR) and IRDye^®^ 700CW Goat anti-Mouse 926–32,210 (1:10,000, LI-COR) secondary antibodies for 1 h at room temperature. After four wash steps with PBS plus 0.1% Tween 20 (PBST), signals were detected using Western Lightning chemiluminescence reagent or analyzed with the Odyssey infrared imaging system and the software program as specified in the Odyssey software manual.

### In-Cell Western Assay

T-47D cells grown at 1×10^3^ cells per well in 96-well plates (Corning Incorporated Costar, #3603). After 24 h incubation, chemical compounds (10 μM) were added to each well. After 48 h incubation, plates were fixed with 4% paraformaldehyde (PFA) for 20min at room temperature (RT). Then the cells were permeabilized with 0.2% Triton X-100 for 15min at RT, and blocked with Odyssey Blocking Buffer (TBS) (LI-COR, 9275000) for 1 h at RT. The cells were incubated at 4°C overnight with a rabbit monoclonal anti-ORF1p antibody (diluted 1:200, Abcam) and a mouse monoclonal anti-beta-actin antibody (diluted 1:250, Abcam) with gentle shakes. After five washes with PBS, the cells were stained with an IRDye^®^ 800RD Goat anti-Rabbit antibody (diluted 1:400, LI-COR, #926–68071) and IRDye^®^ 680CW Goat anti-Mouse antibody (1:600, LI-COR, #926–32210) at RT for 2 h. The microplates, were scanned with the Odyssey system (LI-COR), and the integrated fluorescence intensities representing the protein expression levels were acquired using Image Studio Ver 5.2 software (Egorina et al., 2006). Compared with DMSO control, compounds with an inhibition rate over 50% were defined as promising compounds.

### LINE-1 Dual-Luciferase Assay

This assay is utilized to measure the influence of the compounds on LINE-1 retrotransposition or the L1 5′UTR promoter activity. A 200:1 ratio of FL-L1 or pWA367 and pCMV-Rluc was transfected into HeLa cells, Rluc was served as a control to normalize the Firefly luciferase activity level. Six hours post-transfection, cells were reseeded in 96-well plates, and the plates were incubated for up to 1 day. Next, compounds #1:Hydroxyprogesterone (10μM, 20 μM), # 2: 2,2':5′,2″-Terthiophene (10μM, 20 μM) and # 3: Ethynyl estradiol (10μM, 20 μM) were added. 72–96 h after adding tested compounds to the cells, 25 μl passive lysis buffer was added in each well to lyse cells, and then, 20 μl lysate was detected by dual-luciferase reporter assay system (Promega, USA). Furthermore, the activity levels of Renilla and Firefly luciferase were quantified using Promega Luciferase Assay Kit (Promega) on a Berthold microplate reader.

With respect to test the effects of the compounds or IFNα17 on luciferase gene itself, #1:Hydroxyprogesterone, # 2: 2,2':5′,2″-Terthiophene and # 3: Ethynyl estradiol at the concentration of10 μM or IFNa17 (200U/mL) were added. 48 h post-transfection, cells were lysed and the firefly luciferase activity were measured on a Berthold microplate reader.

### Real-Time RT-PCR

Total RNA was extracted from cells using TRIZOL Reagent (Invitrogen). RNA was reverse transcribed to cDNA using MLV Reverse Transcriptase (Invitrogen) with random primers. The cDNAs were quantified using SsoFast EvaGreen Supermix (Bio-Rad) with Bio Rad iCycler iQ5 Real-Time PCR systems. Primer sequences for cDNAs were as follows: (5′-AAT​GAG​ATC​ACA​TGG​ACA​CAG​GAA​G-3’/5′- TGT​ATA​CAT​GTG​CCA​TGC​TGG​TGC-3′).

To verify the effects of the three compounds or IFNα17, on the transcripts, we selected several key genes for testing. Total RNA was extracted and quantified using qRT-PCR. Primer sequences for cDNAs were as follows:(actin:5′-AGAAAATCTGGCACCACACC-3’/5′-AGAGGCGTACAGGGATAGCA-3′, EIF4G:5′-GTGGACCACAGAACTCCAAGA-3’/5′-AAATAGCCAGACAGCACCCC-3′,MAPK1:5′-CCAGCCCGTCTTGGCTTATC-3’/5′-GAATCTCTCTCTGGTGCGGC-3’; P53: 5′-TGA​CAC​GCT​TCC​CTG​GAT​TG-3’/5′-TCC​GGG​GAC​AGC​ATC​AAA​TC-3′,GAPDH:5′-CCAGGTGGTCTCCTCTGACTTC-3’/5′GTGGTCGTTGAGGGCAATG-3′).

### Cell Proliferation Assay

The 50% cytotoxic concentration (CC_50_) values of 5 drug leads were measured using normal cancer cell line HUVEC and breast cancer cell line T-47D at 6 different inhibitor concentrations. HUVEC and T47-D cell lines in the logarithmic phase were reseeded into 96-well plates (Nest Inc.) at a density of 1×10^3^ cells/well and the plates were incubated for up to 2 days. At the end of each experiment, cells were incubated with 10 µl of the CCK-8 reagent (Vazyme) at 37°C for 2 h. The optical density (OD) at 450 nm was measured by a microplate reader (PerkinElmer Inc.) according to the manufacturer’s instructions. The data are expressed as % of the control. The experiments were repeated at least three times. The 50% effective concentration (EC_50_) of 5 drugs were measured using breast cancer cell line T-47D and 6 different inhibitor concentrations.

To analyze the anti-proliferation effects of the three compounds and IFN17α against carcinoma cells, NCI-H661, NCI-H446, T47-D, MRC-5 and WI-38 cell lines in the logarithmic phase were reseeded into 96-well plates (Nest Inc.) at a density of 1×10^4^ cells/well and the plates were incubated for 24 h. Next, IFN17α (200U/mL) was incubated with NCI-H661, NCI-H446, T47-D and MRC-5 cells, compounds #1(Hydroxyprogesterone), #2 (2,2':5′,2″-Terthiophene) and #3 (Ethynyl estradiol) were added at the concentrations of 10μM and 20 μM respectively, for consecutive 6 days. Cells were incubated with 10 µl of the CCK-8 reagent (Vazyme) each day at 37°C for 2 h. The optical density (OD) at 450 nm was measured by a microplate reader (PerkinElmer Inc.) according to the manufacturer’s instructions. The experiments were repeated at least three times.

## Data Availability

The original contributions presented in the study are included in the article/Supplementary Material, further inquiries can be directed to the corresponding authors.
