# Supplementary material for: A High Throughput Cell-Based Screen Assay for LINE-1 ORF1p Expression Inhibitors Using the In-Cell Western Technique
Source: Front Pharmacol. 2022 May 24;13:881938. doi: 10.3389/fphar.2022.881938 (PMC9171067; doi:10.3389/fphar.2022.881938)
Supplement: Supplementary file 1 [file DataSheet1.PDF]

Supplementary Material

A High Throughput Cell-based Screen Assay for LINE-1 ORF1p Expression Inhibitors Using the In-Cell Western Technique

Yanni Kou[1†], Shujie Wang[1†], Yanjie Ma[1†], Ning Zhang[1], Zixiong Zhang[1], Qian Liu[1], Yang Mao[1], Rui Zhou[1], Dongrong Yi[1], Ling Ma[1], Yongxin Zhang[1], Quanjie Li[1], Jing Wang[1], Jinhui Wang[2], Xile Zhou[3], Chunnian He[4], Jiwei Ding[1*], Shan Cen[1*], Xiaoyu Li[1*]

[1] Institute of Medicinal Biotechnology, Chinese Academy of Medical Sciences & Peking Union Medical College, Beijing, China.

[2] Peking Union Medical College Hospital, Chinese Academy of Medical Sciences & Peking Union Medical College, Beijing, China.

[3] Department of Colorectal Surgery, The First Affiliated Hospital, Zhejiang University, Hangzhou, China.

[4] Institute of Medicinal Plant Development, Chinese Academy of Medical Science, Beijing, China.

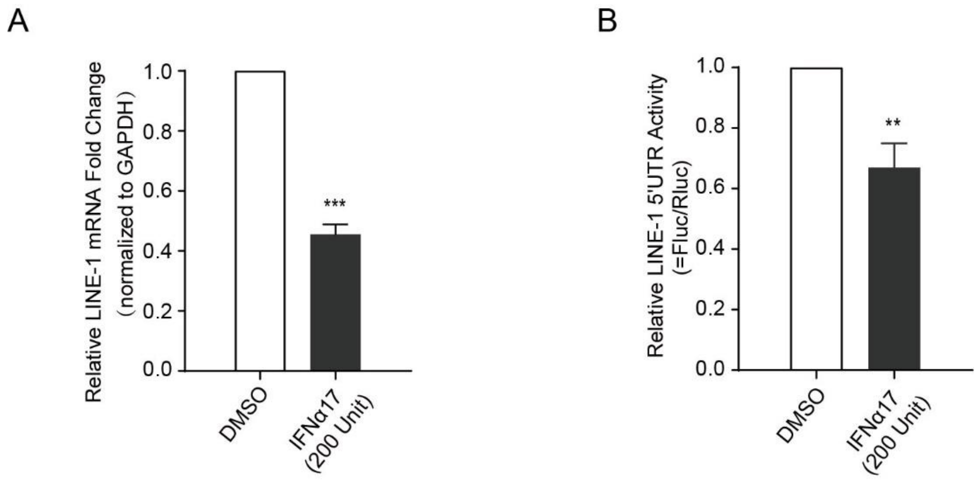

Supplementary Figure 1. The effect of IFNα17 on LINE-1 RNA synthesis. ***(A)***. The effect of IFNα17 on the LINE-1 RNA level in T-47D cells. T-47D cells were treated with IFNα17（200Unit/mL，500Unit/mL） respectively, and 48 h later, the LINE-1 mRNA level was quantified using RT-qPCR. (***B)***. 293 T cells were transfected with L1-FL plasmid, and then treated with the IFNα17（200Unit/mL，500Unit/mL）. The firefly luciferase activity driven by the LINE-1 5’-UTR promoter was measured. P-values were calculated using a standard Student’s t-test. $^{**}p < 0.01$, $^{***}p < 0.001$.

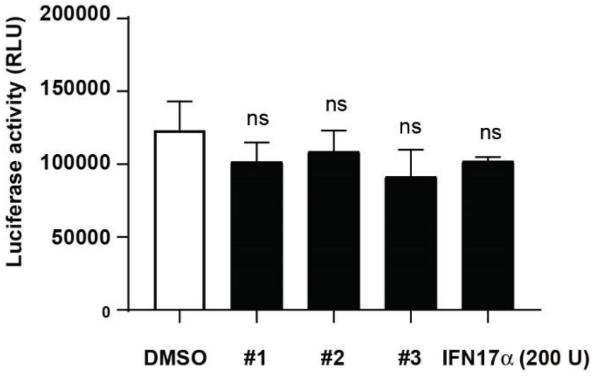

Supplementary Figure 2. The effect of IFNα17 on the luciferase gene. 293 T cells were transfected with the pcDNA4-Luc plasmid, and then treated with the #1, #2 and #3 (10μM) or IFNα17（200Unit/mL）. Two days later, the firefly luciferase activity was measured. P-values were calculated using a standard Student’s t-test. ns, not significant.

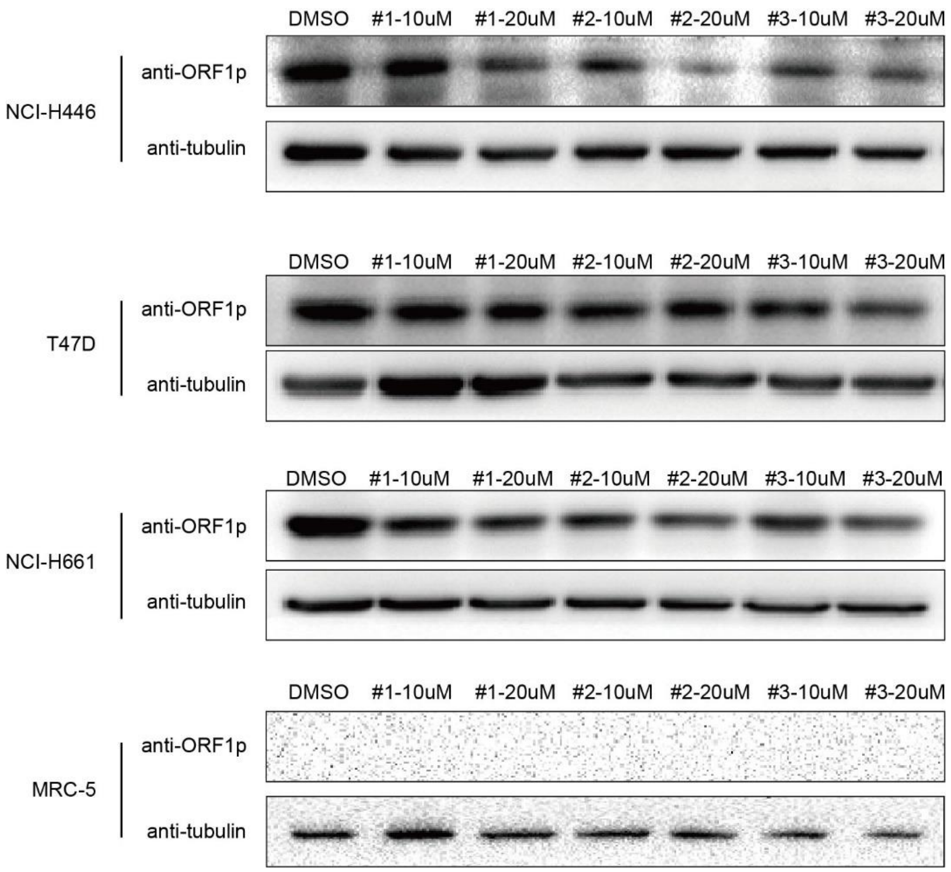

Supplementary Figure 3. The effect of the compounds on ORF1p expression. NCI-H446, NCI-H661, T47D and MRC-5 cells were treated with the #1, #2 and #3 (10μM,20μM), and 48h later, LINE-1 ORF1p expression was analyzed by immunoblotting.

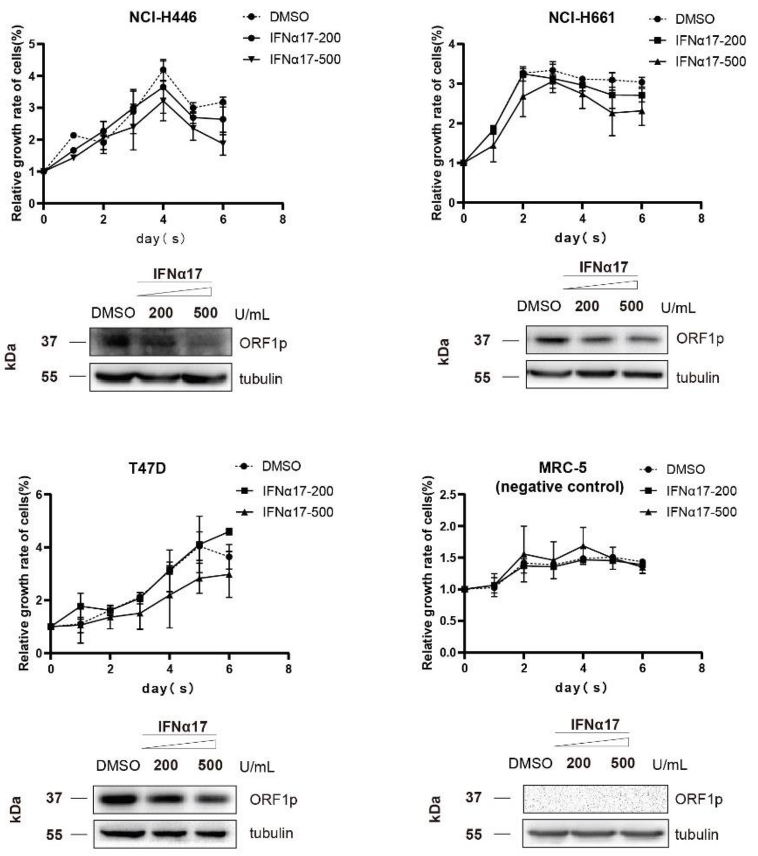

Supplementary Figure 4. The effects of IFNα17 on the proliferation of carcinoma cells. Three carcinoma cells including NCl-H446, NCl-H661 and T47D as well as MRC-5 cell line as a negative control were treated with IFNα17 at the concentration of 200Units/mL or 500Units/mL or DMSO as vehicle control, cell viability is measured for consecutive 6 days by a CCK8 assay. LINE-1 ORF1p expression was analyzed by immunoblotting.

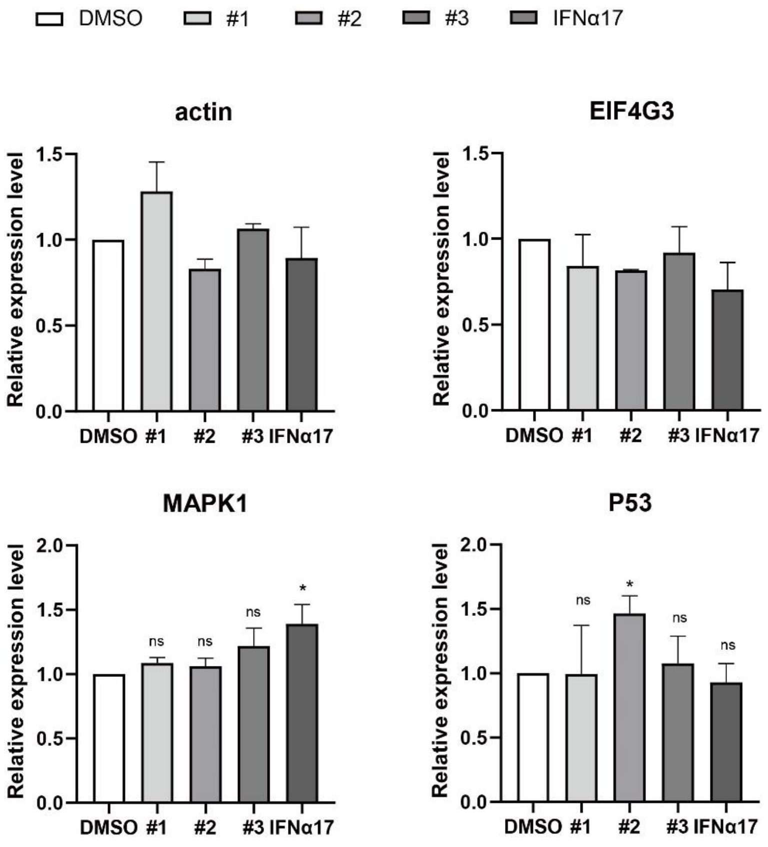

Supplementary Figure 5. The effect of the three compounds on several key cellular genes. T47D cells were treated with 1#, 2# and 3# at the concentration of 10μM or IFNα17 (200Units/mL). 48h later, RNA was extracted, and then an equal amount of RNA was subject to RT-qPCR to measure the expression level of actin, MAPK, EIF4G3 and P53. The data from three independent experiments were summarized in the bar graph. P-values were calculated using a standard Student's t-test. *$p < 0.05$, ns, not significant.
